# AGING COURSES AS A MODEL FOR DEVELOPING PSYCHOLOGICAL LITERACY: INSIGHTS AND ACTIVITIES

**DOI:** 10.1093/geroni/igae098.2694

**Published:** 2024-12-31

**Authors:** Nicole Alea, Carla Strickland-Hughes

**Affiliations:** University of California Santa Barbara, Santa Barbara, California, United States; University of the Pacific, Stockton, California, United States

## Abstract

Psychological literacy refers to understanding psychological content and applying it to: the self, for adaptive self-understanding and management of effective socioemotional goals; local communities, so that students have, for example, the skills to fulfill employer’s needs; and international contexts, so that students have awareness of what is required to become a global citizen. Psychological literacy is relevant for students in all disciplines, as it prepares them for the world after graduation. In this presentation, we suggest that courses with an aging component, like gerontology and psychology of aging, can serve as model courses for developing psychological literacy among students, and should be earmarked as such in undergraduate curriculum. This suggestion will be grounded in three points: (i) aging is self-relevant but feels far-off for traditional-aged undergraduate students; (ii) this means that aging courses naturally tend to showcase the relevance of the material for individuals and society before content to engage students; and (iii) that aging courses tend to emphasize knowledge needed for success in professions and relationships. These three points mean that aging courses encourage psychological literacy. Example course activities, such as activities that create opportunities for intergenerational connections, activities that require exploration of aging careers, and activities that encourage students to propose innovations that will improve life for aging individuals and societies, will be provided to emphasize these points. We will conclude with strategies to include these activities in courses taught in different programs and institutional contexts.

